# Supplementary material for: Central amygdala CRF+ neurons promote heightened threat reactivity following early life adversity in mice
Source: Nat Commun. 2024 Jun 29;15:5522. doi: 10.1038/s41467-024-49828-3 (PMC11217353; doi:10.1038/s41467-024-49828-3)
Supplement: Supplementary file 1 — Supplementary Information [file 41467_2024_49828_MOESM1_ESM.pdf]

Supplementary Figures 1 – 4.

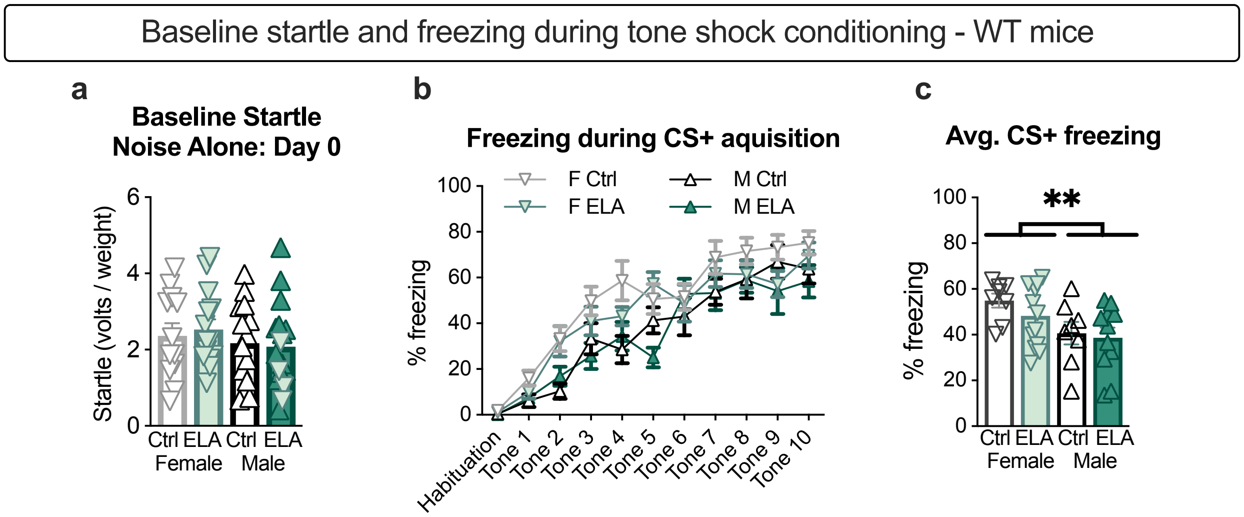

Supplementary Figure 1: Behavioral phenotypes in WT mice.

a) Baseline startle was measured on Day 0, prior to cued-fear conditioning. Baseline startle reactivity to the WN was not impacted by sex, or rearing condition (2-way ANOVA $_{sex}$ $F_{(1, 53)} = 1.25$, $p = 0.26$, $\eta^2 = 0.14$, $\alpha = 0.18$; 2-way ANOVA $_{rearing}$ $F_{(1, 53)} = 0.01$, $p = 0.90$, $\eta^2 = 0.01$, $\alpha = 0.05$). n = 12 (F Ctrl), 14 (F ELA, M Ctrl), 17 (M ELA).

b) Female WT mice exhibit accelerated, and increased conditioned freezing compared to males (3-way RM ANOVA $_{sex\ x\ trial}$ $F_{(10, 360)} = 2.09$, $p = 0.02$, $\eta^2 = 0.22$, $\alpha = 0.98$; 3-way RM ANOVA $_{sex}$ $F_{(1, 36)} = 7.98$ $p = 0.007$, $\eta^2 = 0.38$, $\alpha = 0.99$; 3-way RM ANOVA $_{trial}$ $F_{(6.9, 249.9)} = 81.9$, $p < 0.001$, $\eta^2 = 1.49$, $\alpha = 1.0$). n = 8 (F Ctrl, M Ctrl), 11 (M ELA), 13 (F ELA).

c) Avg. freezing across 10 tones was higher in female mice compared to male (2-way ANOVA $_{sex}$ $F_{(1, 36)} = 7.88$, $p = 0.008$, $\eta^2 = 0.45$, $\alpha = 0.79$). n = 8 (F Ctrl, M Ctrl), 11 (M ELA), 13 (F ELA).

Source data are provided as a Source Data file.

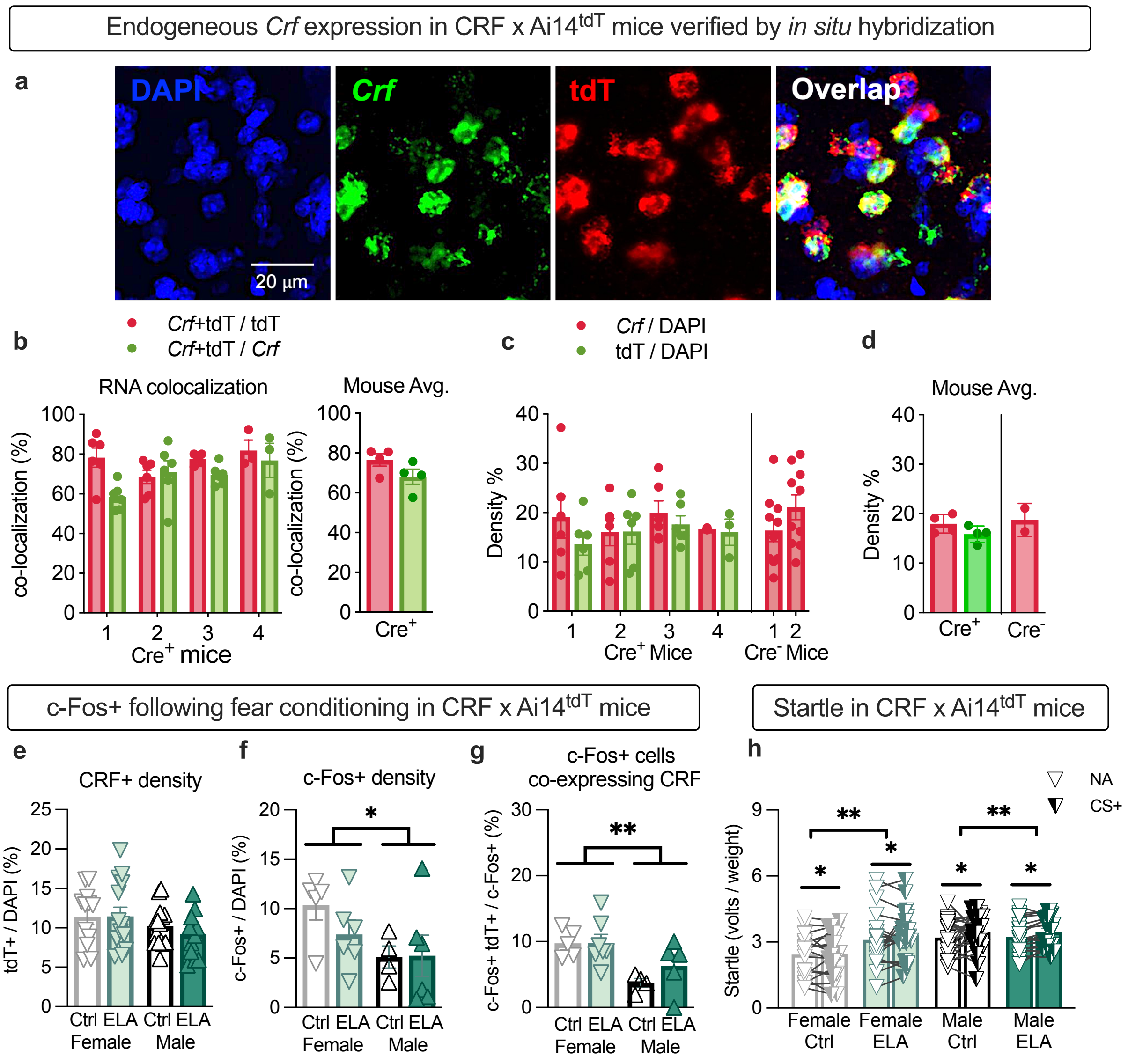

Supplementary Figure 2: Endogenous *Crf* transcript in CRF x Ai14 mice, c-Fos+ expression following fear conditioning and startle phenotype in CRF x Ai14 mice.

a) Representative image of tdTomato (tdT) transcript and endogenous *Crf* transcript in the CRF-ires-Cre::Ai14^{tdTomato} mouse line using RNAscope *in situ* hybridization.
b) Co-expression of tdT and *Crf* in tdT cells (red bars) and co-expression of tdT and *Crf* in *Crf* cells (green bars). *Crf* and tdT expression was counted from the left and right hemisphere of 3 CeAL slices (n=6 ROI) in 4 individual mice. The average colocalization of *Crf* and tdT relative to tdT was 76.49% (Mouse 1: 80.58%, Mouse 2: 67.39%, Mouse 3: 77.35%, Mouse 4: 80.65%). The average colocalization of *Crf* and TdT relative to *Crf* was 68.09% (Mouse 1: 57.63%, Mouse 2: 70.05%, Mouse 3: 68.90, Mouse 4: 75.75).
c) The density of *Crf* transcript relative to DAPI was quantified in 4 Cre+ mice (n=6 slices / mouse; Mouse 1: 19.10%, Mouse 2: 16.07%, Mouse 3: 19.95%, Mouse 4: 16.70%) and in 2 Cre- mice (n=10 slices / mouse; Mouse 1: 16.38%, Mouse 2: 21.13).
d) The average density of endogenous *Crf* transcript expression in the CeAL of Cre+ mice (avg: 17.95%) was not different to that of Cre- mice (avg: 18.74%).

e) The density of CRF+ neurons in the CeAL of CRF x A were not significantly impacted by sex, rearing or a sex by rearing interaction (2-way ANOVA $_{sex}$ $F_{(1, 49)} = 2.14$, $p = 0.14$, $\eta^2 = 0.04$, $\alpha = 0.30$; 2-way ANOVA $_{rearing}$ $F_{(1, 49)} = 0.47$, $p = 0.49$, $\eta^2 = 0.009$, $\alpha = 0.10$; 2-way ANOVA $_{sex\ x\ rearing}$ $F_{(1, 49)} = 0.0003$, $p = 0.57$, $\eta^2 = 0.0006$, $\alpha = 0.05$). n = 12 (F Ctrl, M ELA), 14 (F ELA), 15 (M Ctrl).
f) Percent of c-Fos+ / DAPI following CS+ conditioning was not impacted by sex or ELA rearing (2-way ANOVA $_{sex}$ $F_{(1, 16)} = 3.98$, $p = 0.06$, $\eta^2 = 0.19$, $\alpha = 0.53$; 2-way ANOVA $_{rearing}$ $F_{(1, 16)} = 0.19$, $p = 0.66$, $\eta^2 = 0.01$, $\alpha = 0.07$). n = 5 (F Ctrl), 6 (F ELA, M ELA), 4 (M Ctrl).
g) The percent of c-Fos+ neurons that co-expressed CRF+ was greater in female mice compared to males (2-way ANOVA $_{sex}$ $F_{(1, 16)} = 7.42$, $p = 0.01$, $\eta^2 = 0.31$, $\alpha = 0.80$) and was not impacted by ELA rearing (2-way ANOVA $_{rearing}$ $F_{(1, 16)} = 0.45$, $p = 0.50$, $\eta^2 = 0.02$, $\alpha = 0.09$; 2-way ANOVA $_{sex\ x\ rearing}$ $F_{(1, 16)} = 0.68$, $p = 0.42$, $\eta^2 = 0.04$, $\alpha = 0.13$). n = 5 (F Ctrl), 6 (F ELA, M ELA), 4 (M Ctrl).
h) ELA rearing increased startle exhibited by CRF x Ai14 mice (3-way RM ANOVA $_{rearing}$ $F_{(1, 79)} = 4.14$, $p = 0.04$, $\eta^2 = 0.23$, $\alpha = 0.99$) and was elevated in CS+ trials compared to NA trials (3-way RM ANOVA $_{cue}$ $F_{(1, 79)} = 5.71$, $p = 0.01$, $\eta^2 = 0.06$, $\alpha = 0.62$). n = 19 (F Ctrl, M ELA), 22 (F ELA), 23 (M Ctrl).
Source data are provided as a Source Data file.

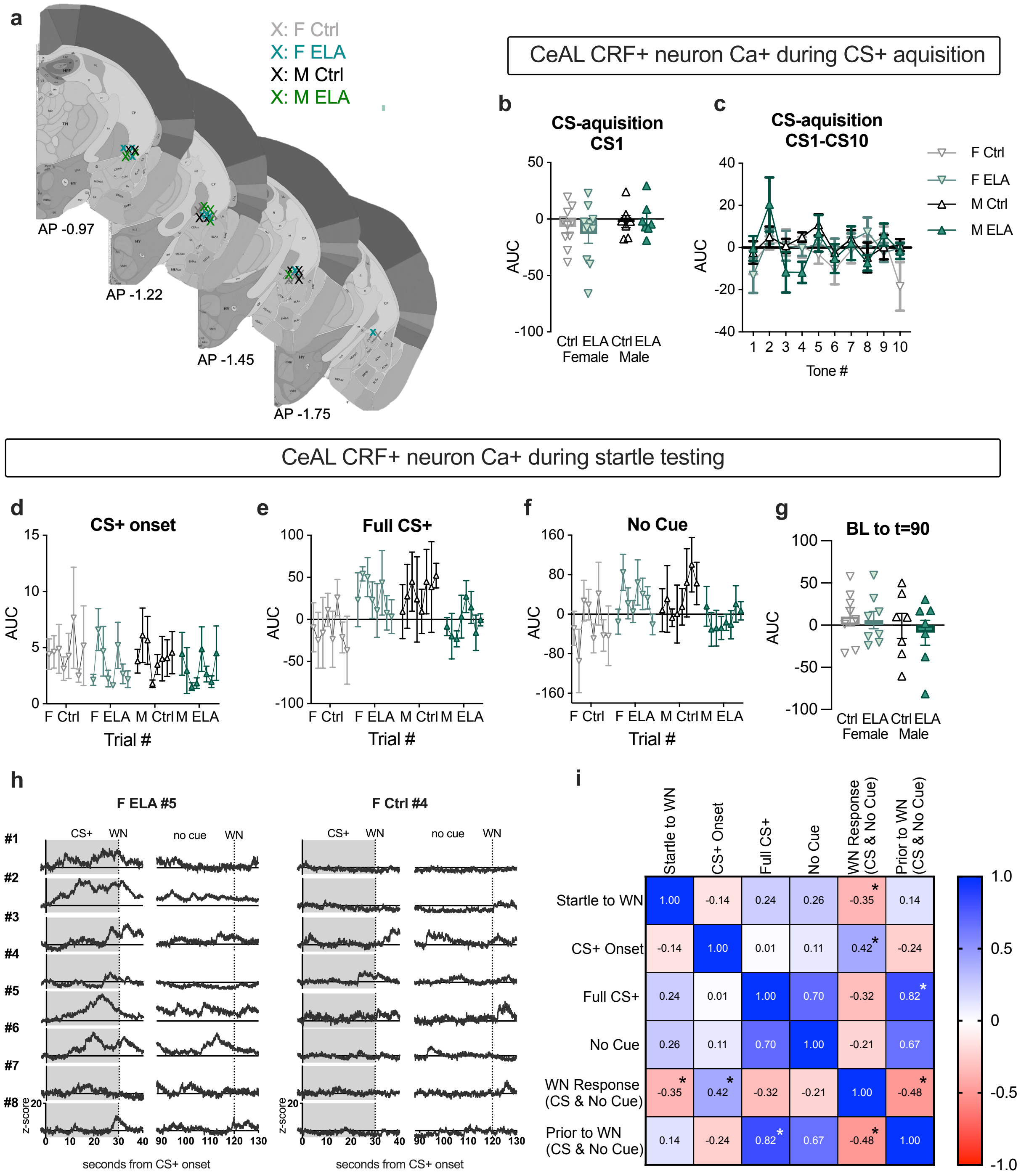

Supplementary Figure 3: CeAL CRF+ neuron Ca+ activity during CS+ acquisition and startle testing

a) Map of estimated fiber tip locations in female Ctrl (grey), female ELA (teal), male Ctrl (black), and male ELA (green) mice.

b) CRF+ CeAL activity in response to the first tone presentation during CS-acquisition. The AUC was calculated on the z-score traces during the 30 sec tone presentations aligned to tone onset. Activity was not influenced by ELA-rearing and did not differ by sex (3-way ANOVA $_{rearing}$: $F_{(1, 30)}$ =

0.09, $p = 0.766$, $\eta^2 = 0.04$, $\alpha = 0.05$; 3-way ANOVA $_{sex}$: $F_{(1, 30)} = 1.579$, $p = 0.218$, $\eta^2 = 0.22$, $\alpha = 0.25$). n=8 (F Ctrl, F ELA), 7 (M Ctrl, M ELA).

c) CeAL CRF+ neuron activity in response to 10 CS+ presentations during CS-acquisition was not impacted by ELA or sex (3-way ANOVA $_{rearing}$: $F_{(1, 30)} = 0.064$, $p = 0.80$, $\eta^2 = 0.01$, $\alpha = 0.05$; 3-way ANOVA $_{sex}$: $F_{(1, 30)} = 1.122$, $p = 0.298$, $\eta^2 = 0.01$, $\alpha = 0.07$). n=8 (F Ctrl, F ELA), 7 (M Ctrl, M ELA).

d) AUC during CS+ onset was not impacted by trial # (3-way ANOVA $_{trial}$: $F_{(3.5, 92.3)} = 1.29$, $p = 0.28$, $\eta^2 = 0.01$, $\alpha = 0.29$) or by ELA rearing (3-way ANOVA $_{rearing}$: $F_{(1, 26)} = 1.53$, $p = 0.22$, $\eta^2 = 0.001$, $\alpha = 0.06$). n=8 (F Ctrl, F ELA), 7 (M Ctrl, M ELA).

e) AUC during full CS+ was not impacted by trial # (3-way ANOVA $_{trial}$: $F_{(5.5, 138.4)} = 0.48$, $p = 0.80$, $\eta^2 = 0.10$, $\alpha = 0.16$) and was differentially impacted by ELA in males and females (3-way ANOVA $_{sex\ x\ rearing}$: $F_{(1, 26)} = 6.06$, $p = 0.02$, $\eta^2 = 0.25$, $\alpha = 0.91$). n=8 (F Ctrl, F ELA), 7 (M Ctrl, M ELA).

f) AUC during the no-cue period normalized to CS+ onset (t = 0) was not impacted by trial # (3-way ANOVA $_{trial}$: $F_{(5.14, 130.1)} = 0.52$, $p = 0.75$, $\eta^2 = 0.10$, $\alpha = 0.10$) and was differentially impacted by ELA in males and females (3-way ANOVA $_{sex\ x\ rearing}$: $F_{(1, 26)} = 5.70$, $p = 0.02$, $\eta^2 = 0.14$, $\alpha = 0.34$). n=8 (F Ctrl, F ELA), 7 (M Ctrl, M ELA).

g) AUC calculated from Ca+ traces normalized to t = 90, to assess CeAL CRF+ neuron activity during the no cue period that was independent of the CS+. AUC was not impacted by sex or rearing condition (2-way ANOVA $_{sex}$: $F_{(1, 26)} = 1.06$, $p = 0.31$, $\eta^2 = 0.17$, $\alpha = 0.15$; 2-way ANOVA $_{rearing}$: $F_{(1, 26)} = 0.22$, $p = 0.63$, $\eta^2 = 0.08$, $\alpha = 0.07$; 2-way ANOVA $_{sex\ x\ rearing}$: $F_{(1, 26)} = 0.02$, $p = 0.87$, $\eta^2 = 0.03$, $\alpha = 0.05$). n=8 (F Ctrl, F ELA), 7 (M Ctrl, M ELA).

h) Example traces from Female ELA #5 and Female Ctrl #4 during 8 presentations of the 30 sec CS+ (t=0-30) co-terminating with the WN (t=30) and the 30 sec no cue period (t=90-120) terminating with the WN (t=120). Z-scores were time-locked to the onset of the CS+ for each trial.

i) Correlation matrix illustrating Spearman R values for the relationship between startle and AUC calculated during CS+ onset, full CS+, no-cue period, response to WN, and prior to WN. Startle to the WN was negatively correlated with Ca+ activity in response to the WN ($R = -0.35$, $p = 0.05$). Ca+ activity to CS+ onset was positively correlated with Ca+ activity in response to the WN ($R = 0.41$, $p = 0.02$). Ca+ activity prior to the WN was positively correlated with Ca+ activity during the full CS+ ($R = 0.82$, $p < 0.0001$). Ca+ activity prior to the WN was negatively correlated with Ca+ activity in response to the WN ($R = -0.48$, $p = 0.006$). n=8 (F Ctrl, F ELA), 7 (M Ctrl, M ELA).
Source data are provided as a Source Data file.

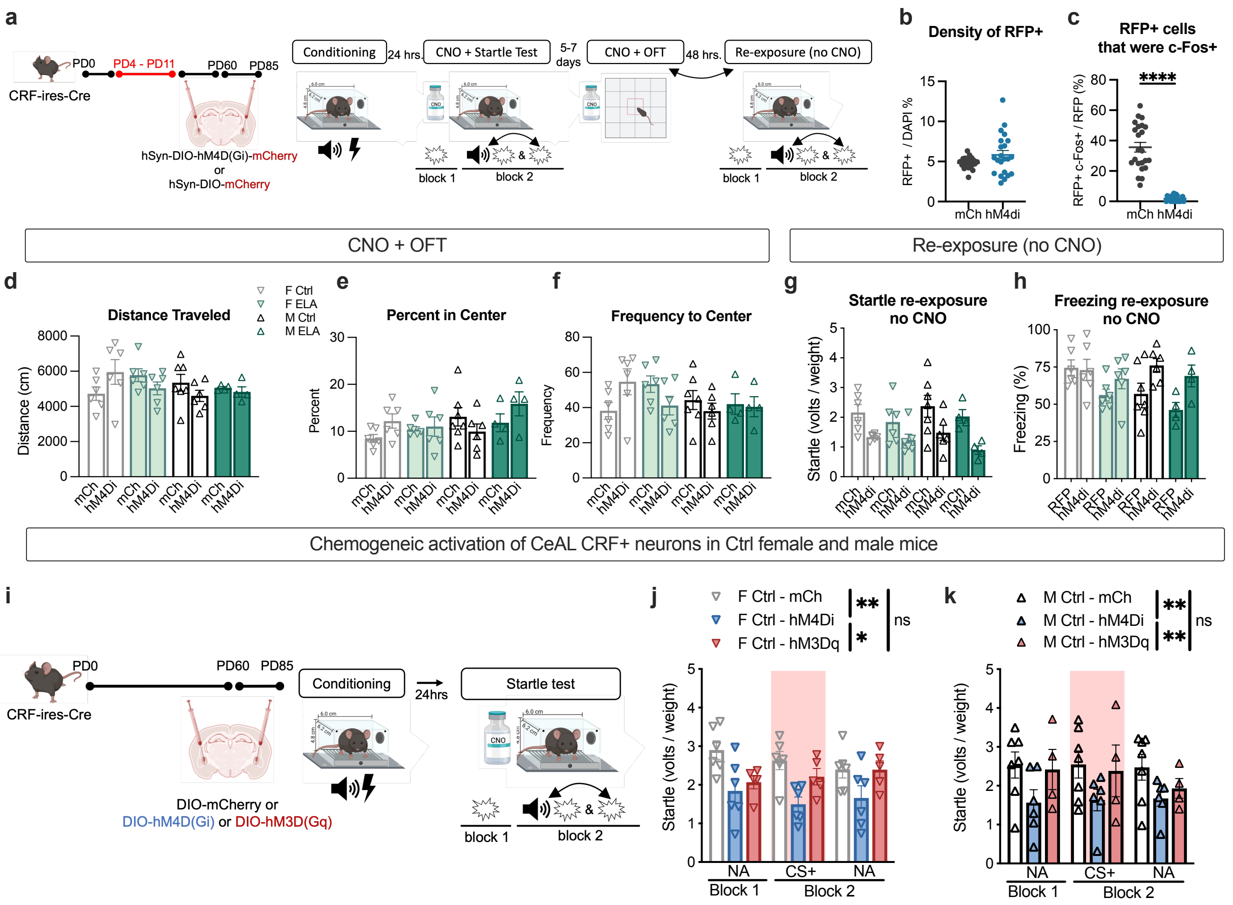

Supplementary Figure 4: Chemogenetic inhibition and activation of CeAL CRF+ neurons

a) Experimental design: Adult male (M) and female (F) CRF-ires-Cre mice reared in control (Ctrl) or early life adversity (ELA) conditions received bilateral CeAL injections of AAV-hSyn-DIO-hM4D(Gi)-mCherry or AAV9-hSyn-DIO-mCherry virus. Clozapine N-oxide (CNO) was administered 30 min prior to startle testing. A week later, mice were exposed to an open field following CNO administration and re-exposure to the startle test without CNO. The OFT and re-exposure test presentations were counterbalanced with 48hrs in between testing days.

b) The density of RFP+ cells were quantified in mCherry- and hM4di-expressing mice to confirm comparable levels of viral expression (Two-tailed Unpaired: t = 1.82, df = 43, p = 0.07, d = 0.53, α = 0.42). n=23 (mCh), 22 (hM4di).

c) Co-expression of RFP+ with c-Fos+ was quantified to measure the percent of CRF+ DREADD-expressing cells that were silenced following CNO-administration. Mice expressing the hM4di virus in CeAL CRF+ neurons exhibited significantly reduced c-Fos+ in RFP+ cells compared to mCherry-expressing control mice (Two-tailed unpaired: t = 10.35, df = 43 p < 0.0001, d = 3.12, α = 1.0). n=23 (mCh), 22 (hM4di).

d) The distance traveled (cm) in the open field was not impacted by virus, sex, or rearing (3-way ANOVA $_{virus}$: $F_{(1, 37)} = 0.15$, p = 0.69, $\eta^2 = 0.06$, α = 0.06; 3-way ANOVA $_{sex}$: $F_{(1, 37)} = 1.68$, p = 0.20, $\eta^2 = 0.21$, α = 0.28; 3-way ANOVA $_{rearing}$: $F_{(1, 37)} = 0.001$, p = 0.96, $\eta^2 = 0.007$, α = 0.05). n=7 (mCh M Ctrl), n= 6(mCh F Ctrl, hM4Di F Ctrl, mCh F ELA, hM4Di F ELA, hM4Di M Ctrl), n=4 (mCh M ELA, hM4Di M ELA).

e) The percent spent in the center of the open field was not impacted by virus, sex, or rearing (3-way ANOVA $_{virus}$: $F_{(1, 37)} = 1.12$, p = 0.29, $\eta^2 = 0.17$, α = 0.20; 3-way ANOVA $_{sex}$: $F_{(1, 37)} = 3.09$, p = 0.08, $\eta^2 = 0.28$,

α = 0.47; 3-way ANOVA $_{rearing}$: $F_{(1, 37)}$ = 1.27, p = 0.26, η^2 = 0.18, α = 0.22). n=7 (mCh M Ctrl), n= 6(mCh F Ctrl, hM4Di F Ctrl, mCh F ELA, hM4Di F ELA, hM4Di M Ctrl), n=4 (mCh M ELA, hM4Di M ELA).

f) The frequency to the center of the open field was not impacted by virus, sex, or rearing (3-way ANOVA $_{virus}$: $F_{(1, 37)}$ = 0.04, p = 0.83, η^2 = 0.03, α = 0.05; 3-way ANOVA $_{sex}$: $F_{(1, 37)}$ = 1.98, p = 0.16, η^2 = 0.23, α = 0.32; 3-way ANOVA $_{rearing}$: $F_{(1, 37)}$ = 0.01, p = 0.90, η^2 = 0.01, α = 0.05). Post-hoc multiple comparisons conducted to explore a virus x sex x rearing interaction (3-way ANOVA virus x sex x rearing: $F_{(1, 37)}$ = 4.36, p = 0.04, η^2 = 0.34, α = 0.61) did not reveal significant differences between groups ($p > 0.05$). n=7 (mCh M Ctrl), n= 6(mCh F Ctrl, hM4Di F Ctrl, mCh F ELA, hM4Di F ELA, hM4Di M Ctrl), n=4 (mCh M ELA, hM4Di M ELA).

g) The avg. startle upon re-exposure to the startle paradigm without CNO administration revealed a main effect of virus (3-way ANOVA $_{virus}$: $F_{(1,27)}$ = 20.64, $p < 0.0001$, η^2 = 0.74, α = 0.99) and was not differentially influence by sex or rearing (3-way ANOVA $_{sex}$: $F_{(1,37)}$ = 0.06, p = 0.80, η^2 = 0.04, α = 0.05; 3-way ANOVA $_{rearing}$: $F_{(1,37)}$ = 3.06, p = 0.08, η^2 = 0.28, α = 0.46). n=7 (mCh M Ctrl), n= 6(mCh F Ctrl, hM4Di F Ctrl, mCh F ELA, hM4Di F ELA, hM4Di M Ctrl), n=4 (mCh M ELA, hM4Di M ELA).

h) The percent freezing upon re-exposure without CNO averaged across trials was influenced by viral expression and rearing but not sex (3-way ANOVA $_{virus}$: $F_{(1,37)}$ = 8.28, p = 0.006, η^2 = 0.47, α = 0.87; 3-way ANOVA $_{rearing}$: $F_{(1,37)}$ = 5.61, p = 0.02, η^2 = 0.38, α = 0.72; 3-way ANOVA $_{sex}$: $F_{(1,37)}$ = 1.61, p = 0.21, η^2 = 0.20, α = 0.27). n=7 (mCh M Ctrl), n= 6(mCh F Ctrl, hM4Di F Ctrl, mCh F ELA, hM4Di F ELA, hM4Di M Ctrl), n=4 (mCh M ELA, hM4Di M ELA).

i) Chemogenetic activation experimental design: Mice received bilateral CeAL injections of AAV-hSyn-DIO-hM4D(Gi)-mCherry, AAV-hSyn-DIO-hM3D(Gq)-mCherry, or AAV9-hSyn-DIO-mCherry virus. The effect of chemogenetic activation or inhibition of CeAL CRF+ neurons in female and male control reared mice was measured during startle testing. Illustration was created using BioRender.com.

j) A main effect of virus (1-way ANOVA $_{virus}$: $F_{(2, 6)}$ = 17.91, p = 0.001, η^2 = 0.46, α =0.87) revealed that startle was significantly lower in control female mice expressing hM4Di virus compared to both mCherry control (p = 0.003) and hM3Dq (p = 0.04) virus. n = 6 (mCh F Ctrl, hM4Di F Ctrl), 5 (hM3Dq F Ctrl).

k) A main effect of virus (1-way ANOVA $_{virus}$: $F_{(2, 6)}$ = 24.14, p = 0.001, η^2 = 0.47, α =0.88) revealed that startle was significantly lower in control male mice expressing hM4Di virus compared to mCherry control (p = 0.001) and hM3D1 (p = 0.009). n = 7 (mCh M Ctrl), 6 (hM4Di M Ctrl), 4 (hM3Dq M Ctrl).

Source data are provided as a Source Data file. Supplemental Figure 4a,i created with BioRender.com released under a Creative Commons Attribution-NonCommercial-NoDerivs 4.0 International license.
