# Supplementary material for: Central amygdala CRF+ neurons promote heightened threat reactivity following early life adversity in mice
Source: Nat Commun. 2024 Jun 29;15:5522. doi: 10.1038/s41467-024-49828-3 (PMC11217353; doi:10.1038/s41467-024-49828-3)
Supplement: Supplementary file 3 — Reporting Summary [file 41467_2024_49828_MOESM3_ESM.pdf]

nature portfolio

Corresponding author(s): Kevin G. Bath

Last updated by author(s): May 13, 2024

Reporting Summary

Nature Portfolio wishes to improve the reproducibility of the work that we publish. This form provides structure for consistency and transparency in reporting. For further information on Nature Portfolio policies, see our Editorial Policies and the Editorial Policy Checklist.

Statistics

For all statistical analyses, confirm that the following items are present in the figure legend, table legend, main text, or Methods section.

n/a	Confirmed	
☐	☒	The exact sample size (*n*) for each experimental group/condition, given as a discrete number and unit of measurement
☐	☒	A statement on whether measurements were taken from distinct samples or whether the same sample was measured repeatedly
☐	☒	The statistical test(s) used AND whether they are one- or two-sided<br>*Only common tests should be described solely by name; describe more complex techniques in the Methods section.*
☐	☒	A description of all covariates tested
☐	☒	A description of any assumptions or corrections, such as tests of normality and adjustment for multiple comparisons
☐	☒	A full description of the statistical parameters including central tendency (e.g. means) or other basic estimates (e.g. regression coefficient) AND variation (e.g. standard deviation) or associated estimates of uncertainty (e.g. confidence intervals)
☐	☒	For null hypothesis testing, the test statistic (e.g. *F*, *t*, *r*) with confidence intervals, effect sizes, degrees of freedom and *P* value noted<br>*Give P values as exact values whenever suitable.*
☒	☐	For Bayesian analysis, information on the choice of priors and Markov chain Monte Carlo settings
☒	☐	For hierarchical and complex designs, identification of the appropriate level for tests and full reporting of outcomes
☐	☒	Estimates of effect sizes (e.g. Cohen's *d*, Pearson's *r*), indicating how they were calculated

Our web collection on statistics for biologists contains articles on many of the points above.

Software and code

Policy information about availability of computer code

Data collection	Startle reflex data were collected using Med Associates Startle Reflex package. Ethovision XT15 (Noldus, US) was used to record videos during startle reflex to track freezing levels and to track animals during open field test. LUX RX10x processor (Tucker Davis Technologies) was used to record fiber photometry data.
Data analysis	Fiber photometry data was processed using the TDT Python Package (Tucker Davis Technologies). GraphPad Prism version 9 was used for statistical analyses.

For manuscripts utilizing custom algorithms or software that are central to the research but not yet described in published literature, software must be made available to editors and reviewers. We strongly encourage code deposition in a community repository (e.g. GitHub). See the Nature Portfolio guidelines for submitting code & software for further information.

Data

Policy information about availability of data

All manuscripts must include a data availability statement. This statement should provide the following information, where applicable:

- Accession codes, unique identifiers, or web links for publicly available datasets
- A description of any restrictions on data availability
- For clinical datasets or third party data, please ensure that the statement adheres to our policy

Source data are provided as a Source Data file.

Research involving human participants, their data, or biological material

Policy information about studies with human participants or human data. See also policy information about sex, gender (identity/presentation), and sexual orientation and race, ethnicity and racism.

Reporting on sex and gender	Not applicable - the current work does not involve human participants.
Reporting on race, ethnicity, or other socially relevant groupings	Not applicable - the current work does not involve human participants.
Population characteristics	Not applicable - the current work does not involve human participants.
Recruitment	Not applicable - the current work does not involve human participants.
Ethics oversight	Not applicable - the current work does not involve human participants.

Note that full information on the approval of the study protocol must also be provided in the manuscript.

Field-specific reporting

Please select the one below that is the best fit for your research. If you are not sure, read the appropriate sections before making your selection.

☒ Life sciences ☐ Behavioural & social sciences ☐ Ecological, evolutionary & environmental sciences

For a reference copy of the document with all sections, see nature.com/documents/nr-reporting-summary-flat.pdf

Life sciences study design

All studies must disclose on these points even when the disclosure is negative.

Sample size	Sample size was determined from previous studies within our lab and power analysis were conducted using G*Power3.0. Sample sizes are listed in the figure legends.
Data exclusions	Data were excluded from mice when the virus location and/or optic fiber placement were not located in the central amygdala.
Replication	The startle phenotype data (greater startle in female ELA mice compared to female Ctrl mice) were replicated multiple times within our lab, including within the Crh-Ai14 and Crh-Cre transgenic mouse lines included in the supplementary material.
Randomization	Mouse dams were randomly assigned to either control or ELA rearing conditions. Mice that underwent DREADD experiments were randomly assigned to each virus group.
Blinding	Experimenters were blinded to the animal groups throughout behavioral experiments and analysis. Cell counts were conducted by an experimenter blind to rearing condition, sex, and viral expression group.

Reporting for specific materials, systems and methods

We require information from authors about some types of materials, experimental systems and methods used in many studies. Here, indicate whether each material, system or method listed is relevant to your study. If you are not sure if a list item applies to your research, read the appropriate section before selecting a response.

Materials & experimental systems

n/a	Involved in the study	
☐	☒	Antibodies
☒	☐	Eukaryotic cell lines
☒	☐	Palaeontology and archaeology
☐	☒	Animals and other organisms
☒	☐	Clinical data
☒	☐	Dual use research of concern
☒	☐	Plants

Methods

n/a	Involved in the study	
☒	☐	ChIP-seq
☒	☐	Flow cytometry
☒	☐	MRI-based neuroimaging

Antibodies

Antibodies used	anti-cfos in rabbit (abcam, cat# 190289), anti-somatostatin in rat (Thermofisher cat# MA5-16987), anti-rabbit 657 cat# ab150079, anti-rat IgG2a FITC conjugate (Thermofisher cat# PA1-84761)
Validation	Antibodies used have been validated by the manufacturers.

Animals and other research organisms

Policy information about studies involving animals; ARRIVE guidelines recommended for reporting animal research, and Sex and Gender in Research

Laboratory animals	C57BL/6N mice, Crh-IRES-Cre (Jax strain# 012704), Crh-IRES-Cre bred with Ai14 (Jax strain # 007914). Mice underwent early life adversity rearing from postnatal day 4-11. All surgeries and behaviors were conducted in adult mice (postnatal day 60-110).
Wild animals	No wild animals were used in this study
Reporting on sex	Both male and female mice were used in this study.
Field-collected samples	No field-collected samples are included in this study.
Ethics oversight	All experimental procedures were approved by Columbia University Institutional Animal Care and Use Committee (IACUC).

Note that full information on the approval of the study protocol must also be provided in the manuscript.

Plants

Seed stocks	Plants were not used in this study
Novel plant genotypes	Plants were not used in this study
Authentication	Plants were not used in this study
